# Hydrogen Sulfide Delivery to Enhance Bone Tissue Engineering Cell Survival

**DOI:** 10.3390/ph17050585

**Published:** 2024-05-02

**Authors:** Soheila Ali Akbari Ghavimi, Trent J. Faulkner, Rama Rao Tata, August J. Hemmerla, Samantha E. Huddleston, Farnoushsadat Rezaei, Ethan S. Lungren, Rui Zhang, Erin E. Bumann, Bret D. Ulery

**Affiliations:** 1 Department of Chemical and Biomedical Engineering, University of Missouri, Columbia, MO 65211, USA; saliakbarighavimi@mgh.harvard.edu (S.A.A.G.); ramarao.tata2@gmail.com (R.R.T.); samanthahuddleston2022@u.northwestern.edu (S.E.H.);; 2 Department of Chemistry, University of Missouri, Columbia, MO 65211, USA; 3 Department of Oral and Craniofacial Sciences, University of Missouri, Kansas City, MO 64110, USA; bumanne@umkc.edu; 4 NextGen Precision Health Institute, University of Missouri, Columbia, MO 65211, USA; 5 Materials Science & Engineering Institute, University of Missouri, Columbia, MO 65211, USA

**Keywords:** bone regeneration, hydrogen sulfide, calcium phosphate, cytoprotection, mesenchymal stem cells

## Abstract

Though crucial for natural bone healing, local calcium ion (Ca^2+^) and phosphate ion (P_i_) concentrations can exceed the cytotoxic limit leading to mitochondrial overload, oxidative stress, and cell death. For bone tissue engineering applications, H_2_S can be employed as a cytoprotective molecule to enhance mesenchymal stem cell (MSC) tolerance to cytotoxic Ca^2+^/P_i_ concentrations. Varied concentrations of sodium hydrogen sulfide (NaSH), a fast-releasing H_2_S donor, were applied to assess the influence of H_2_S on MSC proliferation. The results suggested a toxicity limit of 4 mM for NaSH and that 1 mM of NaSH could improve cell proliferation and differentiation in the presence of cytotoxic levels of Ca^2+^ (32 mM) and/or P_i_ (16 mM). To controllably deliver H_2_S over time, a novel donor molecule (thioglutamic acid—GluSH) was synthesized and evaluated for its H_2_S release profile. Excitingly, GluSH successfully maintained cytoprotective level of H_2_S over 7 days. Furthermore, MSCs exposed to cytotoxic Ca^2+^/P_i_ concentrations in the presence of GluSH were able to thrive and differentiate into osteoblasts. These findings suggest that the incorporation of a sustained H_2_S donor such as GluSH into CaP-based bone graft substitutes can facilitate considerable cytoprotection, making it an attractive option for complex bone regenerative engineering applications.

## 1. Introduction

Bone defects are the second leading cause of disability affecting more than 1.7 billion people worldwide [1]. When bone fractures occur, free radicals and reactive oxygen species (ROS) are generated within the damaged tissue, causing an imbalance between these and antioxidants, damaging cellular macromolecules and altering their functions through oxidative stress [2]. This oxidative stress causes Ca^2+^/P_i_ influx into the cytoplasm from the extracellular environment followed by Ca^2+^/P_i_ passage into the cell mitochondria. The trauma associated with fractures or other major bone injuries also damages the blood supply, resulting in local hypoxia [3]. An increase in intracellular Ca^2+^ levels is a primary response of many cell types to hypoxia [4]. Finally, after considerable bone tissue damage, Ca^2+^/P_i_ concentrations in the blood and urine will decrease [5] while levels local to the defect site will increase, causing the formation of a soft callus which is necessary for bone remodeling [6]. These increases in extracellular Ca^2+^/P_i_ concentrations can cause an intracellular overload [7]. All three of these phenomena contribute to Ca^2+^/P_i_ mitochondrial overload, disrupting and accelerating cellular metabolism leading to cell death [3]. In addition, mitochondrial Ca^2+^/P_i_ overload has been found to lead to greater ROS production [4] which further increases oxidative stress leading to even greater intracellular Ca^2+^/P_i_ concentrations.

While bones are capable of remodeling themselves, reconstruction of critical-sized bone defects is challenging as intervention is required to facilitate adequate healing [8]. Since the body’s natural reaction to bone tissue damage is to locally increase Ca^2+^/P_i_ concentrations, many regenerative engineering approaches leverage these ions as signaling molecules in their design. Bioactive ceramics, especially calcium phosphates (CaPs) and calcium sulfate (CaS) composites have been widely used in bone regeneration because of their similarity to native bone mineral content [9,10]. Once immersed in an aqueous solution, CaPs undergo dissolution and precipitation as the result of ion transfer at the solid–liquid interface yielding a net release of calcium ions (Ca^2+^) and phosphate ions (H_2_PO^4−^, HPO_3_^2−^, or PO_4_^3−^-P_i_) from the material [11]. These ions have been found to facilitate bone regeneration [12,13]. Ca^2+^ and P_i_ osteoinductivity exists in a concentration range termed the therapeutic window where enough ions are present to facilitate stem cell osteogenic differentiation, but not too many to overwhelm the cells, inducing their death. Our previous efforts have shown that Ca^2+^ concentrations of 32 mM or more and P_i_ concentrations of 16 mM or more are cytotoxic for mesenchymal stem cells in vitro [14] and are the limits that can be used for biomaterials-based bone regeneration engineering applications [15,16].

Improving cell tolerance to increased Ca^2+^/P_i_ concentrations can lead to a broader and enhanced therapeutic window by limiting the cytotoxic effects of high concentration Ca^2+^/P_i_. One promising option to mitigate ion-mediated toxicity is hydrogen sulfide (H_2_S), a gasotransmitter simple signaling molecule [17] that has been found capable of suppressing oxidative stress in mitochondria [18,19,20] as well as MSC survival and proliferation under high stress conditions like hypoxia, oxidative stress, and serum deprivation [21,22]. Unfortunately, there are only a few H_2_S donors commercially available, with most research having to employ the burst release kinetics achieved by sodium hydrosulfide (NaHS) or sodium sulfide (Na_2_S) [23]. Recent efforts have focused on the development of H_2_S donors that offer more sustained release kinetics. Some H_2_S donors have been developed, including GYY4137 for more sustained and hydrolytic cleavage, but the toxicity and clearance of the remaining small molecule backbone after H_2_S release has yet to be studied [24,25]. Zhou and colleagues developed H_2_S donors through thioacid substitution in amino acids (i.e., glycine and valine) to improve the compatibility, predictability, and degradation kinetics of a H_2_S donor as a cardioprotective reagent [24]. While promising, the rapid release rate and lack of a conjugatable chemical group makes thioglycine and thiovaline difficult to incorporate into various biomaterials for sustained, localized H_2_Sa release to supplement site-directed Ca^2+^/P_i_ osteoinductivity. To address this issue, we have designed and synthesized a novel H_2_S donor with a conjugatable carboxylic acid (i.e., thioglutamic acid—GluSH) and evaluated its cytoprotective effect on mesenchymal stem cells exposed to cytotoxic Ca^2+^/P_i_ concentrations in vitro.

## 2. Results and Discussion

### 2.1. H_2_S Cytotoxicity

**Figure 1:**
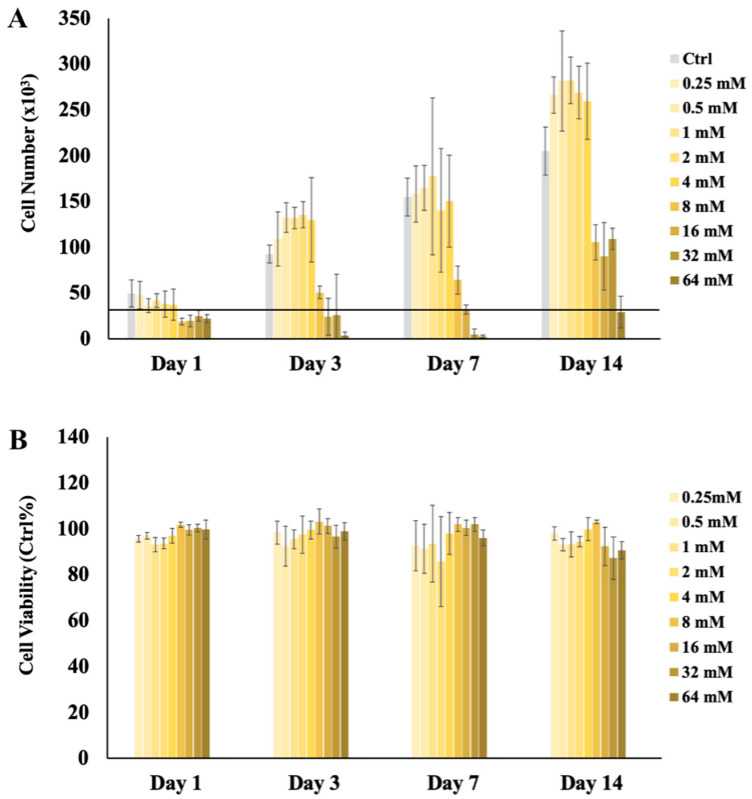
Proliferation and viability of MSCs exposed to different concentrations of NaSH supplemented media. (**A**) Cell count was measured by the Quanti-iT PicoGreen Assay over fourteen days of cell culture in media supplemented with 0–64 mM NaSH for seven days and then culture media with 0 mM NaSH for the next seven days. The black line indicates the original cell seeding number (i.e., 30,000). (**B**) An indirect measure of cell metabolism and viability was determined by NAD(P)H activity over fourteen days using a MTS assay for MSCs exposed to 0–64 mM NaSH for seven days and then culture media with 0 mM NaSH for the next seven days. Error bars indicate ± 1 standard deviation (*n* = 4). Statistical analysis of the data is available in the supplementary information (Tables S1 and S2).

Proliferation and viability of MSCs exposed to different concentrations of NaSH are shown in Figure 1. MSCs seeded on tissue cultured plastic (Ctrl) and incubated in growth media increased to seven times initial cell seeding number over 14 days and cells exposed to concentrations of 4 mM NaSH or less expanded to more than eight times initial cell seeding number (Figure 1a). The cell number for those exposed to 0.5 and 1 mM of NaSH were statistically significantly higher than the control group at day 14 (Table S1). On the other hand, proliferation of MSCs exposed to 8 mM NaSH or more were statistically significantly lower than those provided growth medium supplemented with 0–4 mM NaSH (Table S1). The mildly mitogenic behavior of H_2_S at lower concentrations is likely due to its ability to increase intracellular levels of cyclic guanosine monophosphate (cGMP) [26], which is known to stimulate stem cell proliferation [27]. H_2_S can also inhibit cytochrome c oxidase activity and cause oxidative stress [28] or even directly cause radical-associated DNA damage [29], which is likely responsible for the cell death found at higher H_2_S concentrations.

The results of the MTS assay show that MSCs exposed to 4 mM NaSH or less were over 90% as viable as the control group through 14 days. Interestingly, MSCs subjected to 8 mM NaSH or more were still more than 80% viable as the control group through 14 days (Figure 1b). Taken with the proliferation results, these data indicate that though high concentrations of NaSH adversely affect MSC proliferation, the surviving cells are highly metabolically active over the 14 days of the study.

#### Cytoprotective Effect of H_2_S at High Ca^2+^ and/or P_i_ Concentrations

The cytotoxicity evaluation of NaSH revealed that high NaSH concentration (i.e., 8 mM or more) can decrease cell proliferation. Since there was no statistically significant difference between cell proliferation and viability of the MSCs exposed to 0.25 to 4 mM NaSH, the rest of this research explored the cytoprotective effect of 1 mM NaSH (NaSH_1_) when co-delivered with higher concentrations of Ca^2+^ and/or P_i_. Our previous research indicates that media supplemented with more than 16 mM Ca^2+^ and/or 8 mM P_i_ can be cytotoxic, adversely affecting MSC proliferation and viability [14]. Therefore, 32 mM Ca^2+^ (C_32_—cytotoxic concentration), 16 mM P_i_ (P_16_—cytotoxic concentration), 1 mM NaSH (NaSH_1_—non-cytotoxic concentration), and combinations of Ca^2+^, P_i_, and NaSH_1_ were used to explore the cytoprotective effect of hydrogen sulfide in the initial presence of excess calcium and phosphate ions. After seven days of high concentration exposure, MSCs were exposed to inductive and non-cytotoxic Ca^2+^ (i.e., Ca_16_) and P_i_ (i.e., P_8_) concentrations without NaSH from day seven to day fourteen to better mimic the conditions within the bone fracture site.

**Figure 2:**
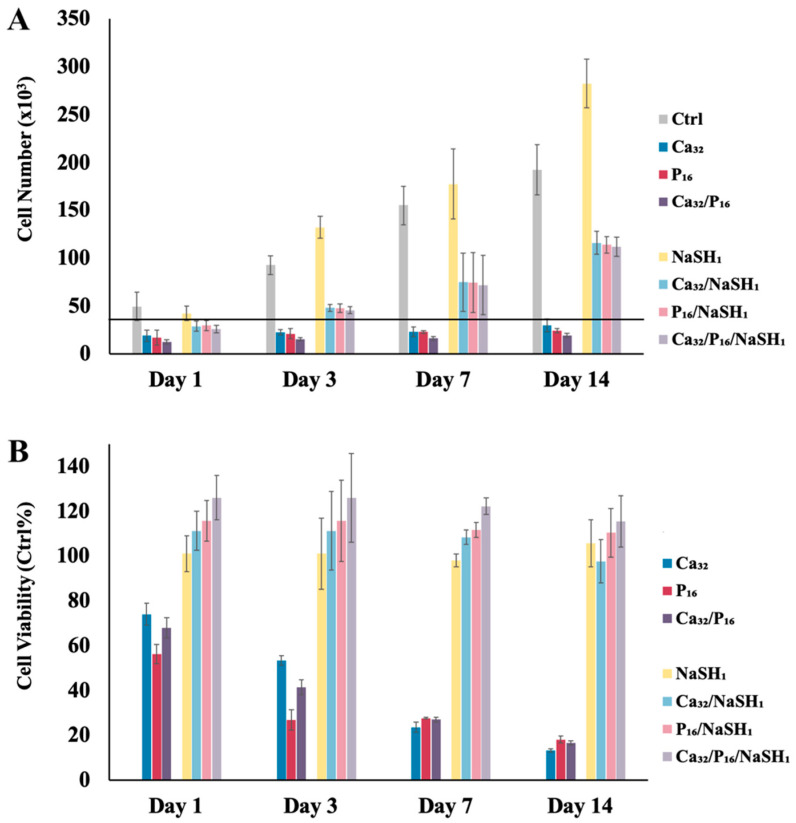
Proliferation and viability of MSCs exposed to different combinations of Ca^2+^-, P_i_-, and/or NaSH-supplemented media. (**A**) Cell count was measured by the Quanti-iT PicoGreen Assay over 14 days of cell culture in media supplemented with no signaling molecules (Ctrl), 32 mM Ca^2+^ (Ca_32_), 16 mM P_i_ (P_16_), Ca_32_/P_16_, 1 mM NaSH (NaSH_1_), Ca_32_/NaSH_1_, P_16_/NaSH_1_, or Ca_32_/P_16_/NaSH_1_ for the first 7 days. This was followed by the cells being exposed for the next seven days to no signaling molecules or half the Ca^2+^ and/or P_i_ concentrations (i.e., Ca_16_ and/or P_8_) they were originally cultured in. The black line indicates the original cell seeding number (i.e., 30,000). (**B**) An indirect measure of cell metabolism and viability was determined by NAD(P)H activity over 14 days using an MTS assay. MSCs were exposed to Ca_32_, P_16_, and/or NaSH_1_ and combination of these molecules (Ca_32_/P_16_, Ca_32_/NaSH_1_, P_16_/NaSH_1_, and Ca_32_/P_16_/NaSH_1_) for the first seven days followed by the aforementioned decrease in Ca^2+^ and/or P_i_ concentrations for the respective groups. Error bars indicate ± 1 standard deviation (*n* = 4). All data were normalized against the results determined for MSCs given non-supplemented media. Statistical analysis of the data is available in the supplementary information (Tables S3 and S4).

Proliferation and viability of cells exposed to different combinations of ionic and gasotransmitter signaling molecules is demonstrated in Figure 2. This is likely due to the cytotoxic effects and the possible osteoinductivity of high Ca^2+^ and P_i_ concentrations (Figure 2A). However, when the MSCs were also supplied with NaSH_1_, their proliferation was statistically significantly greater through 14 days (Table S3). This result demonstrates the positive effect of NaSH on the proliferation of cells exposed to cytotoxic levels of Ca^2+^ and P_i_. It is known that increases in intracellular Ca^2+^ promote mitochondrial calcium uptake, resulting in the loss of mitochondrial membrane potential which is interpreted as a danger signal initiating apoptosis [30]. The addition of NaSH_1_ as a H_2_S donor can help regulate cytosolic Ca^2+^ levels and mitochondrial calcium uptake [31], resulting in higher cell survival compared to MSCs exposed to a high Ca^2+^ concentration alone. Higher P_i_ concentrations can dysregulate the mitochondrial permeability transition pore (MPTP), initiating a pro-apoptotic cascade [32] or further enhancing ongoing apoptosis [33]. By reducing oxidative stress, H_2_S indirectly protects the mitochondria from becoming damaged and activating pro-apoptotic signaling pathways [34].

Cell viability results reveal that the metabolic activity of MSCs exposed to the Ca_32_, P_16_, and Ca_32_/P_16_ were statistically significantly lower than Ca_32_/NaSH_1_ and P_16_/NaSH_1_, and Ca_32_/P_16_/NaSH_1_ through 14 days (Figure 2B and Table S3). High Ca^2+^ and/or P_i_ concentrations can even lead to viability less than 20% when compared to the control group whereas supplementing these same groups with NaSH_1_ preserved cell viability to over 90% of control. To investigate viability, the MTS assay was used which measures cell viability by indirectly determining NAD(P)H-dependent mitochondrial dehydrogenase activity essential to cell metabolism and proliferation oxidation/reduction reactions [35]. The previously mentioned mitochondrial dysregulation due to Ca_32_ and P_16_ that limited cell proliferation understandably negatively impacted cell dehydrogenase activity as well. Overall, NaSH_1_ exhibits a modest mitogenic effect, while cells treated with Ca_32_/NaSH_1_, P_16_/NaSH_1_, or Ca_32_/P_16_/NaSH_1_ at days 7 and 14 show lower proliferative capacity though maintain comparable metabolic activity to control cells. In contrast, cells exposed to cytotoxic levels of calcium and/or phosphate ions (Ca_32_, P_16_, or Ca_32_/P_16_) display reduced cell numbers and diminished viability over time. These results are unsurprising, as rapidly expanding cells do not always show much of an increase in overall metabolic activity and differentiating cells (i.e., likely those exposed to Ca_32_, P_16_, or Ca_32_/P_16_ that were also in the presence of cytotoxicity-mitigating NaSH_1_) often have high metabolic activity on an individual cell basis compared to dividing cells.

**Figure 3:**
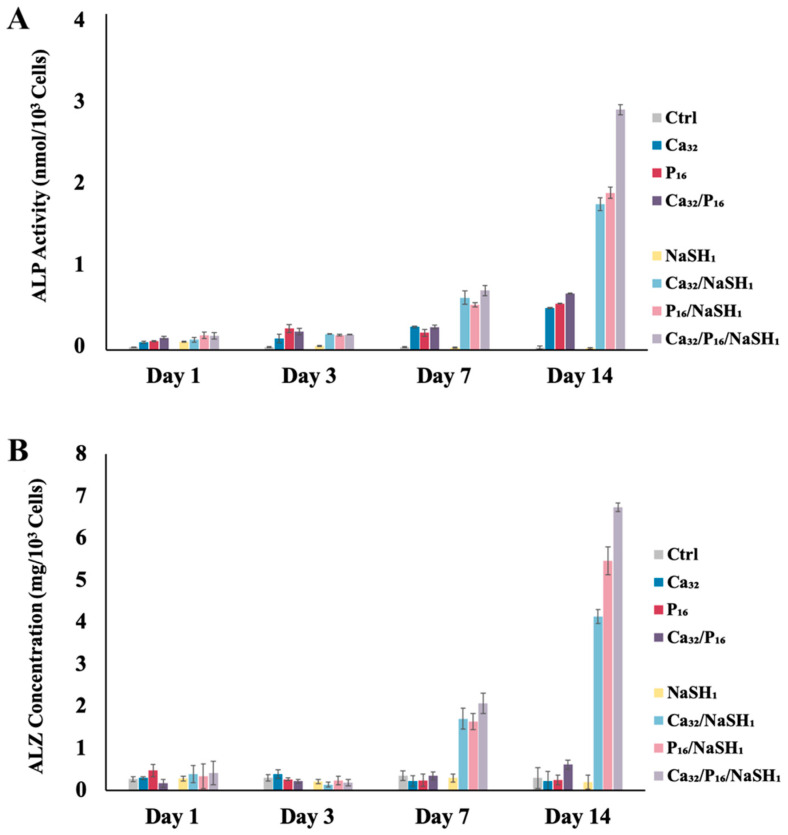
ALP activity and cell-based mineralization of MSCs exposed to different combinations of Ca^2+^-, P_i_-, and/or NaSH-supplemented media. (**A**) Maturation enzyme activity was analyzed by an ALP Assay Kit over 14 days of cell culture in media supplemented with no signaling molecules (Ctrl), 32 mM Ca^2+^ (Ca_32_), 16 mM P_i_ (P_16_), Ca_32_/P_16_, 1 mM NaSH (NaSH_1_), Ca_32_/NaSH_1_, P_16_/NaSH_1_, or Ca_32_/P_16_/NaSH_1_ for the first seven days. This was followed by the cells being exposed for the next seven days to no signaling molecules or half the Ca^2+^ and/or P_i_ concentrations (i.e., Ca_16_ and/or P_8_) they were originally cultured in. (**B**) Alizarin red (ALZ) staining was used as an indirect measure of mineralization over 14 days of cell culture. MSCs were exposed to no signaling molecules (Ctrl), Ca_32_, P_16_, and/or NaSH_1_, and combination of these molecules (Ca_32_/P_16_, Ca_32_/NaSH_1_, P_16_/NaSH_1_, Ca_32_/P_16_/NaSH_1_) for the first seven days followed by the aforementioned decrease in Ca^2+^ and/or P_i_ concentrations for the respective groups. Both ALP activity and ALZ mineralization data were normalized on a per cell basis. Error bars indicate ± 1 standard deviation (*n* = 4). Statistical analysis of the data is available in the supplementary information (Tables S3 and S4).

ALP activity and cell-based mineralization of MSCs cultured with Ca_32_ or P_16_ with or without NaSH_1_ are described in Figure 3. MSC osteogenic differentiation is known to proceed through three stages: proliferation, maturation, and mineralization. ALP, one of the first enzymes expressed during osteogenesis, helps guide bone formation and calcification [36,37]. Specifically, as MSCs proliferate and differentiate into osteoblasts, they secrete ALP, which provides a high local phosphate concentration at the osteoblast surface in preparation for the bone mineralization process [38]. The ALP results presented were normalized by cell number to focus on cell differentiation independent of proliferation. MSCs exposed to media (Ctrl) and media supplemented with NaSH_1_ showed background levels of ALP expression while cells subjected to Ca_32_, P_16_, Ca_32_/P_16_, Ca_32_/NaSH_1_, P_16_/NaSH_1_, and Ca_32_/P_16/_NasSH_1_ showed statistically significantly increased levels of ALP expression compared to the control (Figure 3A and Table S3). Also, cells cultured with Ca_32_ and P_16_ supplemented with NaSH_1_ (i.e., Ca_32_/NaSH_1_, P_16_/NaSH_1_, and Ca_32_/P_16/_NasSH_1_) showed statistically significantly higher levels of ALP expression compared to those without NaSH_1_ (i.e., Ca_32_, P_16_, and Ca_32_/P_16_) at each time point assessed (Figure 3A, Tables S3 and S4). The low ALP activity in MSCs exposed to Ca_32_ and/or P_16_ alone is likely due to their diminished viability, which when modulated by NaSH_1_ can improve overall ALP activity considerably. It has recently been reported that H_2_S can also promote osteoblast differentiation at sites of bone regeneration by triggering deposition of the inorganic mineral matrix and promoting expression of osteogenic genes in human MSCs [36]. However, our results did not show improved ALP activity after exposure of MSCs to NaSH_1_ alone compared to the control group over the course of 14 days, suggesting H_2_S can assist other inductive molecules (i.e., Ca^2+^ and P_i_) though may not be osteoinductive in its own right.

In comparison to the early-stage, non-mineralized matrix deposition process guided by ALP, matrix mineralization is the hallmark of late-stage MSC osteogenesis [39,40]. In this research, acellular experiments were conducted in parallel using the same conditions allowing their results to be subtracted from the complementary cellular groups to eliminate solution-mediated mineralization. These cell-based mineralization results were then normalized by cell number to evaluate improved osteoinductivity by NaSH_1_ independent of its influence on proliferation. Evaluation of MSC mineralization revealed that Ca_32_/NaSH_1_, P_16_/NaSH_1_, and Ca_32_/P_16_/NaSH_1_ induced statistically significantly greater mineral deposition over time (Table S4) as well as greater than cells exposed to Ctrl and NaSH_1_ treatments (Figure 3B and Table S3). Ca_32_, P_16_, and Ca_32_/P_16_ were found to limit mineralization instead of enhancing it over the Ctrl group over time (Figure 3B, Tables S3 and S4) likely due to the toxicity of these treatments. The mineralization results were in agreement with the ALP activity data supporting the beneficial effects of H_2_S when supplementing high concentrations of osteoinductive ions.

### 2.2. H_2_S Release from GluSH

**Figure 4:**
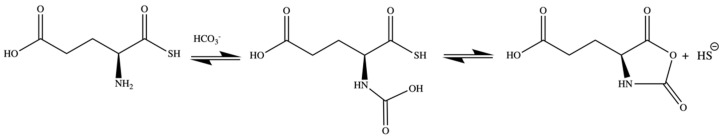
Sulfanyl ion (HS^−^) release mechanism from GluSH in the presence of bicarbonate. The binding of the carboxyl group to the amine creates an unstable complex, which results in cyclizing of the α-amino acid groups into an N-carboxyanhydride, releasing HS^−^. Both reaction steps are reversible and the progression towards HS^−^ release only occurs in the presence of a sufficient bicarbonate concentration [39,41].

**Figure 5:**
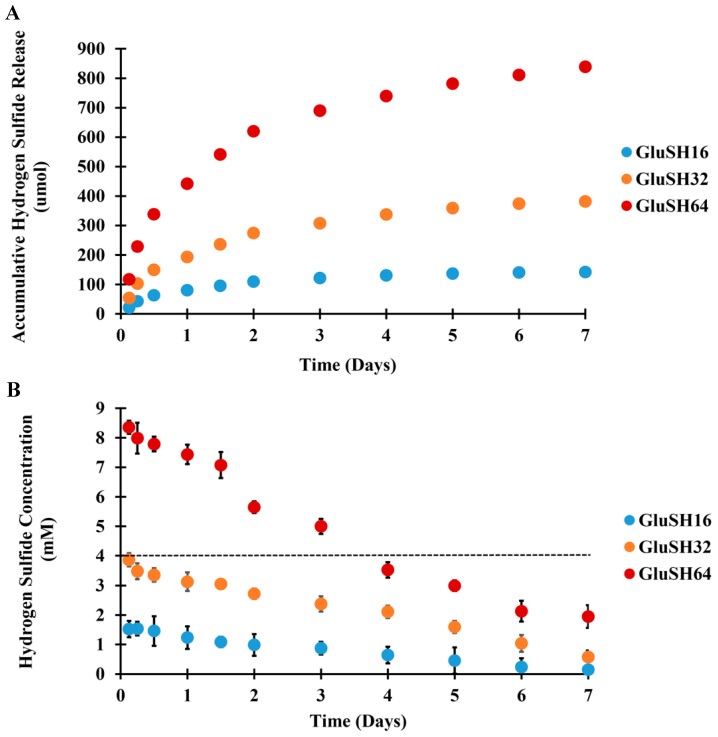
GluSH H_2_S release. (**A**) Accumulative H_2_S release and (**B**) H2S concentration release profiles from 16, 32, and 64 mM GluSH immersed in bicarbonate-supplemented Hank’s Balanced Salt Solution (HBSS) over seven days at 37 °C. Error bars indicate ± 1 standard deviation (*n* = 4).

In order to investigate the cytoprotective effects of sustained H_2_S delivery, GluSH was synthesized and tested for its ability to controllably release H_2_S over time. GluSH is a highly water-soluble amino acid derivative that releases a sulfanyl ion (HS^−^) in the presence of bicarbonate by the chemical reaction outlined in Figure 4 which can then be protonated in water to yield H_2_S [41]. Excitingly, HS^−^ is only produced from GluSH in the presence of bicarbonate, which is a molecule that can be readily found in the human body [40]. Therefore, bicarbonate-supplemented pH and osmotic balancing HBSS was used to investigate H_2_S release from GluSH over time. Varied GluSH concentrations were tested to determine the maximum allowable concentration that maintained H_2_S release below the previously NaHS-determined cytotoxic limit (i.e., 4 mM). Figure 5 summarizes the results of H_2_S production from 16, 32, and 64 mM GluSH over seven days.

As shown in Figure 5A, GluSH released around 50% of its total H_2_S generating payload within the first day. H_2_S continued to be generated through day five, after which little more was released from GluSH. When the data were converted to H_2_S concentration (Figure 5B), 32 mM GluSH was found to maximally release H_2_S payload below the cytotoxic limit (i.e., 4 mM) for seven days.

### 2.3. GluSH-Mediated Stem Cell Proliferation and Differentiation

**Figure 6:**
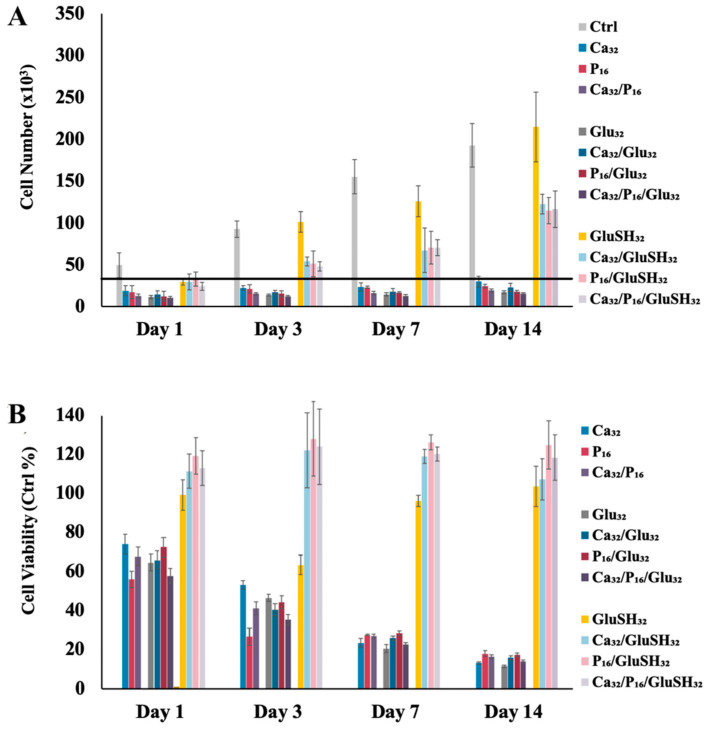
Proliferation and viability of MSCs exposed to different combinations of Ca^2+^-, P_i_-, and/or GluSH-supplemented media. (**A**) Cell count was measured by the Quanti-iT PicoGreen Assay over 14 days of cell culture in media supplemented with no signaling molecules (Ctrl), 32 mM Ca^2+^ (Ca_32_), 16 mM P_i_ (P_16_), 32 mM glutamic acid (Glu_32_), 32 mM thioglutamic acid (GluSH_32_), or combination of these molecules (i.e., Ca_32_/P_16_, Ca_32_/Glu_32_, P_16_/Glu_32_, Ca_32_/P_16_/Glu_32_, Ca_32_/GluSH_32_, P_16_/GluSH_32_, Ca_32_/P_16_/GluSH_32_) for the first 7 days. This was followed by the cells being exposed for the next seven days to no signaling molecules or half the Ca^2+^ and/or P_i_ concentrations (i.e., Ca_16_ and/or P_8_) they were originally cultured in. The black line indicates the original cell seeding number (i.e., 30,000). (**B**) An indirect measure of cell metabolism and viability was determined by NAD(P)H activity over 14 days using an MTS assay. MSCs were exposed to Ca_32_ and/or P_16_ with or without Glu_32_ or GluSH_32_ for the first seven days followed by the aforementioned decrease in Ca^2+^ and/or P_i_ concentrations for the respective groups. All data were normalized against the results determined for MSCs given non-supplemented media. Error bars indicate ± 1 standard deviation (*n* = 4). Statistical analysis of the data is available in the supplementary information (Tables S5 and S6).

To evaluate GluSH bioactivity, MSCs were exposed to GluSH_32_ supplemented with toxic levels of Ca^2+^ and P_i_. To distinguish the difference between the effect of H_2_S release from the rest of the GluSH molecule, glutamic acid (Glu)-supplemented media was also investigated as a control. The proliferation and viability of cells exposed to media supplemented with excess Ca^2+^ and P_i_ with or without Glu or GluSH is summarized in Figure 6. Proliferation of MSCs exposed to Ca^2+^ and/or P_i_ in the presence of GluSH (i.e., Ca_32_/GluSH_32_, P_16_/GluSH_32_, and Ca_32_/P_16_/GluSH_32_) was statistically significantly greater than that of comparative ones supplemented with Ca^2+^ and/or P_i_ alone or with Glu (i.e., Ca_32_, P_16_, Ca_32_/P_16_, Ca_32_/Glu_32_, P_16_/Glu_32_, and Ca_32_/P_16_/Glu_32_) and lower than media-only control and GluSH_32_ (Figure 6A and Table S5). These results are due to the synergistic effect of H_2_S moderating high concentration Ca^2+^ and P_i_ cytotoxicity while allowing these molecules to likely carry out their osteoinductive effects. Interestingly, cells exposed to GluSH_32_ alone proliferated to eight times of their initial seeding concentration demonstrating similar cell population growth kinetics as the control (Figure 6A and Table S5). However, MSCs exposed to Glu_32_ alone did not proliferate over the course of 14 days. These results suggest that the acidic nature of glutamic acid could potentially cause some cytotoxicity [40]. In contrast, GluSH releasing HS^−^ produces the byproduct glutamate N-carboxyanhydride (NCA) (Figure 5), which generates a less acidic solution than glutamic acid while the presence of H_2_S would also serve as a cytoprotectant. The presence of the glutamate NCA byproduct had a mild, transient, negative impact on cell viability at day three (~60%), but this was alleviated by day seven with no effect on cell proliferation indicating the biocompatibility of our novel molecule. Similar to the NaSH data, these cell proliferation and metabolic results are expected as rapidly expanding cells do not always show much of an increase in overall metabolic activity and differentiating cells (i.e., likely those exposed to Ca_32_, P_16_, or Ca_32_/P_16_ that were also in the presence of cytotoxicity-mitigating GluSH_32_) often have high metabolic activity on an individual cell basis compared to dividing cells. The viability of the MSCs exposed to different combinations of signaling molecules reveals that the cells exposed to Ca_32_ and P_16_ as well as Glu were less than 30% metabolically active as those in the control group by day 14 (Figure 6B and Table S5). Supplementing high Ca^2+^ and/or P_i_ concentration media with GluSH mitigated any cytotoxic effects, increasing cell viability to more than 100% as compared to the control group.

**Figure 7:**
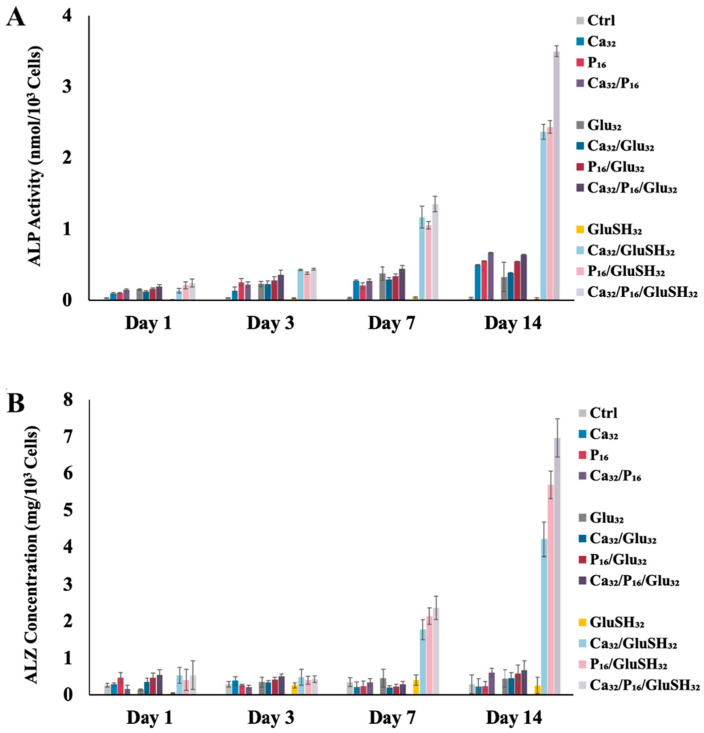
ALP activity and cell-based mineralization of MSCs exposed to different combinations of Ca^2+^-, P_i_-, and/or GluSH-supplemented media. (**A**) Maturation enzyme activity was analyzed by an ALP Assay Kit over 14 days of cell culture in media supplemented with no signaling molecules (Ctrl), 32 mM Ca^2+^ (Ca_32_), 16 mM P_i_ (P_16_), 32 mM glutamic acid (Glu_32_), 32 mM thioglutamic acid (GluSH_32_), or combination of these molecules (i.e., Ca_32_/P_16_, Ca_32_/Glu_32_, P_16_/Glu_32_, Ca_32_/P_16_/Glu_32_, Ca_32_/GluSH_32_, P_16_/GluSH_32_, and Ca_32_/P_16_/GluSH_32_) for the first 7 days. This was followed by the cells being exposed for the next seven days to no signaling molecules or half the Ca^2+^ and/or P_i_ concentrations (i.e., Ca_16_ and/or P_8_) they were originally cultured in. (**B**) Alizarin red (ALZ) staining was used as an indirect measure of mineralization over 14 days of cell culture MSCs were exposed to Ca_32_ and/or P_16_ with or without Glu_32_ or GluSH_32_ for the first 7 days followed by the aforementioned decrease in Ca^2+^ and/or P_i_ concentrations for the respective groups. Both ALP activity and ALZ mineralization data were normalized on a per cell basis. Error bars indicate ± 1 standard deviation (*n* = 4). Statistical analysis of the data is available in the supplementary information (Tables S5 and S6).

The ALP activity and cell-based mineralization of MSCs cultured with Ca_32_ and/or P_16_ with or without GluSH_32_ or Glu_32_ is described in Figure 7. MSCs exposed to non-supplemented media (Ctrl) or media supplemented with Glu_32_ or GluSH_32_ showed background levels of ALP expression while cells subjected to Ca_32_, P_16_, Ca_32_/P_16_, Ca_32_/GluSH_32_, P_16_/GluSH_32_, and Ca_32_/P_16_/GluSH_32_ showed statistically significantly increased levels of ALP expression compared to the control (Figure 7A, Table S5). Cells cultured with Ca_32_ and P_16_ with GluSH_32_ (i.e., Ca_32_/GluSH_32_, P_16_/GluSH_32_, and Ca_32_/P_16_/GluSH_32_) showed statistically significantly higher levels of ALP expression compared to those without GluSH_32_ (i.e., Ca_32_, P_16_, and Ca_32_/P_16_) that also increased over time (Figure 7A, Tables S5 and S6). Assessment of MSC mineralization revealed that Ca_32_/GluSH_32_, P_16_/GluSH_32_, and Ca_32_/P_16_/GluSH_32_ induced statistically significant mineral deposition over time (Table S6) that was greater than all other treatments from day seven onward (Figure 7B and Table S5). MSCs exposed to Ca_32_, P_16_, or Ca_32_/P_16_ showed limited changes over time (Table S6) which while possessing greater mineralization than the Ctrl had a statistically insignificant difference compared to cells exposed to Glu_32_ (Figure 7B and Table S5). While GluSH excitingly widens the differentiative Ca^2+^ and/or P_i_ therapeutic window that leads to increased ALP activity and potentiates mineralization, these studies alone do not fully demonstrate its cell survival benefits within an in vivo environment. When transitioning from in vitro studies, GluSH will need to be functionalized into a physical scaffold for delivery of its components. As such, future studies will focus on the development of polymeric and bio-based scaffolding to controllably release GluSH in critical-sized defect models [40]. These studies would be carried out in comparison to commercially available hydrogen sulfide donors, such as GYY4137, and would assess the biocompatibility of scaffolds, toxicity of hydrogen sulfide donors, H_2_S release kinetics, localized ALP expression, and mineralization and regeneration of bone defects.

## 3. Material and Methods

### 3.1. Preparation of H_2_S, Ca^2+^, and P_i_ Containing Media

H_2_S stock solution (pH = 7.4) was prepared by dissolving 512 mM of NaSH (Sigma-Aldrich, St. Louis USA) in distilled, deionized water (ddH_2_O) at 37 °C. Ca^2+^ stock solution (pH = 7.4) was prepared by dissolving 512 mM calcium chloride (CaCl_2_; Sigma-Aldrich), 25 mM 4-(2-Hydroxyethyl)piperazine-1-ethanesulfonic acid (HEPES; Sigma-Aldrich), and 140 mM sodium chloride (NaCl; Sigma-Aldrich) in ddH_2_O at 37 °C. P_i_ stock solution (pH = 7.4) was prepared by dissolving disodium hydrogen phosphate dihydrate (Na_2_HPO_4_•2H_2_O; Sigma-Aldrich) and sodium dihydrogen phosphate dihydrate (NaH_2_PO_4_•2H_2_O; Sigma-Aldrich) at a ratio of 4:1 Na_2_HPO_4_/NaH_2_PO_4_, 25 mM HEPES, and 140 mM NaCl in ddH_2_O at 37 °C. These stock solutions were made fresh for each experiment and sterilized by 0.22 µm syringe filter. Individual solution concentrations of 0.25, 0.5, 1, 2, 4, 8, 16, 32, and 64 mM H_2_S, 32 mM Ca^2+^, and 16 mM P_i_, as well as combined solution concentrations of 32 mM:1 mM Ca^2+^:H_2_S, 16 mM:1 mM P_i_:H_2_S, and 32 mM:16 mM:1 mM H_2_S:Ca^2+^:P_i_:H_2_S were prepared by diluting stock solutions in growth media consisting of Dulbecco’s modified Eagle’s medium (DMEM, Invitrogen, Carlsbad,CA, USA) supplemented with 10% fetal bovine serum (FBS, Invitrogen) and 1% Penicillin-streptomycin (Pen-Strep, Invitrogen).

### 3.2. Cell Culture and Seeding

Murine mesenchymal stem cells (MSCs) were purchased from Cyagen and initially cultured in T-75 cell culture flasks (Corning, Kennebunk, ME, USA) in growth medium at 37 °C in a humidified incubator supplemented with 5% CO_2_. Media were changed every 48 h until cells approached ~80% confluency after which they were dissociated using a 0.05% trypsin-EDTA (Invitrogen) solution. Detached MSCs were counted by hemocytometer and passed to new T-75 flasks at a splitting ratio of 1:4 or 1:5 dependent on cell count. After the 5th passage, cells were used for in vitro bioactivity studies. Tissue-cultured polystyrene 24-well plates (Corning) were seeded with 30,000 cells/well and exposed to growth media alone as a negative control or growth media containing 0.25, 0.5, 1, 2, 4, 8, 16, 32, or 64 mM NaSH, or 32 mM Ca^2+^ (Ca_32_), or 16 mM P_i_ (P_16_). Additional studies utilized growth media containing 32 mM:16 mM Ca^2+^:P_i_ (Ca_32_/P_16_), 32 mM glutamic acid (Glu_32_), 32 mM GluSH (GluSH_32_), 32 mM:1 mM Ca^2+^:NaSH (Ca_32_/NaSH_1_), 16 mM:1 mM P_i_:NaSH (P_16_/NaSH_1_), 32 mM:16 mM:1 mM Ca^2+^:P_i_:NaSH (Ca_32_/P_16_/NaSH_1_), 32 mM:32 mM Ca^2+^:Glu (Ca_32_/Glu_32_), 16 mM:32 mM P_i_:Glu (P_16_/Glu_32_); 16 mM:32 mM:32 mM Ca^2+^:P_i_:Glu (Ca_32_/P_16_/Glu_32_), 32 mM:32 mM Ca^2+^:GluSH (Ca_32_/GluSH_32_), 16 mM:32 mM P_i_:GluSH (P_16_/GluSH_32_); or 32 mM:16 mM:32 mM Ca^2+^:P_i_:GluSH (Ca_32_/P_16_/GluSH_32_) loaded into a Transwell membrane insert (Corning). MSCs were cultured with these solutions for up to 7 days at 37 °C in a humidified incubator supplemented with 5% CO_2_ and the media containing various compounds were added fresh every 2 days. After day 7, cells exposed to high Ca^2+^ and P_i_ concentrations were treated with 16 mM Ca^2+^ (Ca_16_), 8 mM P_i_ (P_8_), or 16 mM:8 mM Ca^2+^:P_i_ (Ca_16_/P_8_) based on their group without any additional H_2_S releasing molecules with the media containing appropriate ion concentrations changed every 2 days. Cell proliferation, viability, alkaline phosphatase (ALP) activity, and mineralization were assessed at 1, 3, 7, and 14 days.

### 3.3. Proliferation Assay

Cell proliferation was determined using the Quanti-iT PicoGreen dsDNA Assay (Thermo Fisher Scientific, Waltham, MA, USA). At each endpoint, the samples were rinsed with phosphate buffered saline (PBS) and exposed to 1% Triton X-100 (Sigma-Aldrich) followed by three freeze-thaw cycles in order to lyse the cells. Lysates were diluted with TE buffer (200 mM Tris-HCL, 20 mM EDTA, pH 7.5) and mixed with PicoGreen reagent according to the manufacturer’s protocol. A BioTek Cytation 5 fluorospectrometer plate reader was utilized to measure the fluorescence of each sample (ex. 480 nm, em. 520 nm) and the cell number was calculated using a MSC standard curve (0–200,000 cells/mL).

### 3.4. Viability Assay

Cell viability was evaluated at each time point using an MTS Cell Proliferation Colorimetric Assay Kit (BioVision, Cambridge, UK). MTS reagent (20 µL) was added to growth media (500 µL) followed by 4 h incubation at 37 °C in a humidified incubator supplemented with 5% CO_2_. Absorbance of each sample was measured at 490 nm using a plate reader. Cell viability was reported as the ratio of absorbance in the experimental groups compared to the growth media negative control.

### 3.5. Alkaline Phosphatase Activity Assay

Cell ALP activity was quantified at each time point using an Alkaline Phosphatase Assay Kit (BioVision). In brief, 20 µL of cell lysate was combined with 50 µL of p-nitrophenyl phosphate (pNPP) solution in assay buffer. The mixture was incubated for 1 h at room temperature away from light. The reaction was stopped by adding 20 µL of the stop solution and the absorbance of the solution was measured at 405 nm using a plate reader. To eliminate any background effects, 1% Triton X-100 was incubated with pNPP, exposed to stop solution in assay buffer after 1 h, and its absorbance deducted from sample absorbance. The absorbance was converted to content of dephosphorylated p-nitrophenyl (pNP) using a pNP standard curve (0–20 nmol/mL) which was dephosphorylated using excess ALP Enzyme. ALP activity was reported as the pNP content normalized by cell count.

### 3.6. Mineralization Assay

Cell-based mineral deposition was measured using an Alizarin red assay. At each time point, the media were removed after which the cells were washed with ddH_2_O and fixed in 70% ethanol for 24 h. The ethanol was removed and the samples were incubated in 1 mL of 40 mM Alizarin Red solution (Sigma-Aldrich) for 10 min. The samples were rinsed with ddH_2_O several times to make sure all non-absorbed stain was removed. Absorbed Alizarin Red was desorbed using 1 mL of a 10% cetylpyridinium chloride (CPC, Sigma-Aldrich) solution after which stain concentration was measured at 550 nm using a plate reader. Absorbance of each sample was converted to the concentration of absorbed Alizarin Red using a standard curve (0–0.2740 mg/mL). Samples above the standard curve linear range were diluted with CPC solution until a reading in the linear range was obtained. Cell-based mineral deposition was calculated by subtracting mineralization found in blank wells exposed to the same experimental conditions. All results were normalized by cell count.

### 3.7. GluSH Synthesis

**Scheme 1:**
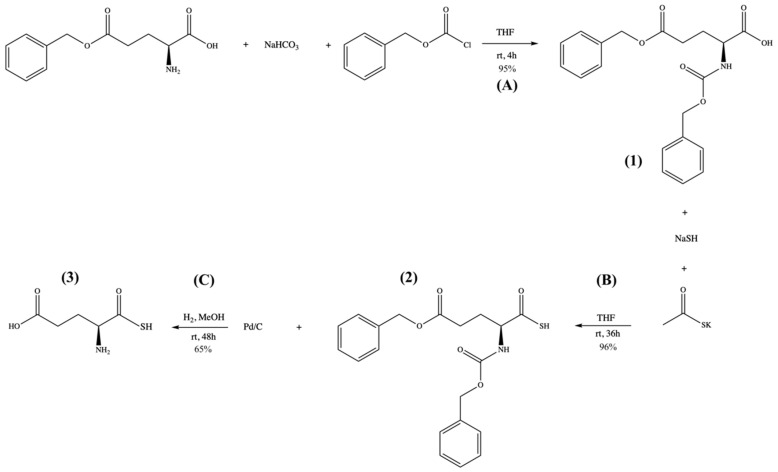
GluSH synthesis process.

The three-step synthesis process is detailed in Scheme 1.

***Reaction A***. Sodium bicarbonate (NaHCO_3_; Sigma-Aldrich) (1.5 mmol) was dissolved in water (50 mL) at room temperature after which 10 mL of dry tetrahydrofuran (THF; Sigma-Aldrich) was added. L-glutamic acid 5-benzyl ester (Alfa Aesar) (4.2 mmol) was dissolved in the reaction mixture followed by dropwise addition of benzyl chloroformate (Sigma Aldrich) (6.5 mL). After 4 h stirring under an argon atmosphere, the reaction mixture turned to a clear solution. To remove the unreacted benzyl chloroformate, the reaction mixture was washed with diethyl ether (60 mL × 2). The aqueous layer was then acidified (pH ~ 2) with 1 mM hydrochloric acid (HCl). The mixture was then extracted with ethyl acetate (60 mL × 2), dried over anhydrous sodium sulfate (Na_2_SO_4_), and evaporated under reduced pressure using a rotary evaporator (Buchi). The final product, N-benzyloxycarbonyl-L-glutamic acid 5-benzyl ester **(1)**, was a white powder and used without further purification. This product was dissolved in deuterated chloroform for ^1^H-NMR spectroscopy analysis (Figure S1): ^1^H NMR (500 MHz, CDCl_3_) δ 7.35–7.27 (m, 10H), 5.56–5.54 (d, 1H), 5.11 (s, 4H), 4.43–4.40 (m, 1H), 2.51–2.45 (m, 2H), 2.27–2.24 (m, 1H), 2.04 (m, 1H).

***Reaction B.*** N-benzyloxycarbonyl-L-glutamic acid 5-benzyl ester (**(1)**, 10 mmol) was dissolved in 15 mL of dry THF in a round bottom flask. The solution was then treated with NaSH (20 mmol) and thioacetic acid (10 mmol) at room temperature. After the reaction mixture was stirred in open air for 48 h, the solvent was evaporated under reduced pressure using a rotary evaporator. The residue was diluted with water (50 mL) and acidified with 1 mM HCl (pH ~ 2). This aqueous solution was extracted with ethyl acetate (30 mL × 3). The combined organic layer was washed with brine, dried over anhydrous Na_2_SO_4_, and evaporated under reduced pressure using a rotary evaporator. The final product was purified using a silica gel column chromatography and the pure N-benzyloxycarbonyl-L-thioglutamic acid 5-benzyl ester **(2)** was eluted at 40% ethyl acetate in hexane as a yellow oil. This product was dissolved in deuterated chloroform for ^1^H-NMR spectroscopy analysis (Figure S2): ^1^H NMR (500 MHz, CDCl_3_) δ 7.35–7.27 (m, 10H), 5.53–5.51 (d, 1H), 5.12 (s, 4H), 4.46–4.45 (m, 1H), 2.56–2.48 (m, 2H), 2.31–2.29 (m, 1H), 2.07 (m, 1H).

***Reaction C.*** N-benzyloxycarbonyl-L-thioglutamic acid 5-benzyl ester (**(2)**, 10 mmol) was dissolved in methanol (40 mL) after which 10 wt% palladium on carbon (Pd/C; Sigma-Aldrich) was added to the mixture as a catalyst for the hydrogenation reaction. The reaction flask was sealed and vacuum purged prior to the addition of hydrogen gas. The reaction was stirred for 48 h under hydrogen at room temperature. The reaction mixture was then dissolved in water and vacuum filtered. The clear filtrate was then evaporated under reduced pressure using a rotary evaporator which left a yellow powder of L-thioglutamic acid (GluSH, **(3)**). This product was dissolved in deuterium oxide for ^1^H-NMR spectroscopy analysis (Figure S3): ^1^H NMR (500 MHz, D_2_O) δ 3.96–3.79 (t, 1H), 2.57–2.56 (m, 2H), 2.16 (m, 2H).

### 3.8. H_2_S Release Study

H_2_S release from GluSH was measured in ddH_2_O at 37 °C over 7 days. GluSH loaded into a Transwell membrane insert (Corning) was placed in centrifuge tubes containing Hank’s buffer saline solution (HBSS) supplemented with excess amounts of bicarbonate (1.5 mol per 1 mol GluSH). Each centrifuge tube was sealed with vacuum glue and parafilm. At certain time points, 1 mL of the release solution was extracted from the reaction tube using a needle and replaced with 1 mL of HBSS and bicarbonate solution. The solutions were then immediately assayed to determine their H_2_S concentration using a fluorescent method measuring the conversion of monobromobimane (MBB) to thiobimane [41]. Briefly, dibromobimane (Sigma-Aldrich) was dissolved in HBSS at a concentration of 500 µM and incubated with the test samples at room temperature for 5 min after which florescence (ex. 340 nm, em. 465 nm) was measured. A standard curve was created by dissolving different concentrations of NaSH in the 500 µM dibromobimane in HBSS solution, incubating at room temperature for 5 min, and measuring the florescence. The concentration of the H_2_S at each time point was then calculated by comparing the test sample values to the NaSH standard curve.

### 3.9. Statistical Analysis

JMP software (version 12) was used to make comparisons between groups with Tukey’s HSD test specifically utilized to determine pairwise statistical differences (*p* < 0.05). The statistical analysis results are reported in the supporting information section. Groups that possess different letters have statistically significant differences in mean whereas those that possess the same letter have means that are statistically insignificant in their differences.

## 4. Conclusions

This research evaluated the cytoprotective effect of H_2_S on MSCs experiencing osteoinductive ion overload similar to what can occur within the bone defect site microenvironment. It was determined that an effective H_2_S therapeutic range (<4 mM) exists that can improve cell proliferation and differentiation even in the presence of cytotoxic levels of calcium and phosphate ions. Furthermore, the novel thioglutamic acid was developed through glutamic acid modification that sustained H_2_S release at therapeutic levels for seven days, which provided effective mitigation of ion-mediated calcium and phosphate cytotoxicity while maintaining their inherent osteoinductive capacity. These results support the considerable promise of thioglutamic acid as a useful product in helping to facilitate cell survival in critical sized bone defects.

**Figure 8:**
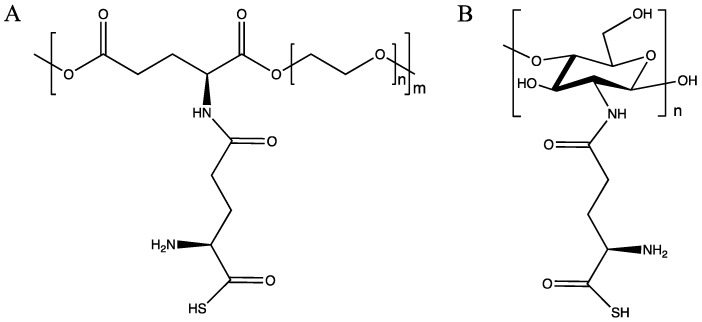
GluSH incorporation into GluGluSH-PEG polyester (**A**) and attachment to chitosan biopolymer (**B**).

Future work will include the covalent attachment of thioglutamic acid with another glutamic acid to create glutamylglutamic acid (GluGluSH), a diacid monomer. This monomer can be readily polymerized with various diols (e.g., PEG) to generate a series of bioactive polyesters (Figure 8A), all of which can be utilized to enable sustained and localized release of hydrogen sulfide. Another alternative is the attachment of thioglutamic acid to an existing natural biopolymer through its carboxylic acid to the amine group of chitosan (Figure 8B). These options allow for future materials characterization that will seek to optimize H_2_S release at physiological conditions along with analyzing the impact of the companion metabolic products from these degradable polymer systems both in vitro and in vivo. Hydrogels formed from these have the potential to achieve localized in vivo hydrogen sulfide release to couple with an expanded range of calcium and phosphate ions for bone regenerative engineering applications.

## Data Availability

Data is contained within the article and Supplementary Material.

## Supplementary Materials

The following supporting information can be downloaded at: https://www.mdpi.com/article/10.3390/ph17050585/s1, Figure S1: NMR spectrum of N-benzyloxycarbonyl-L-glutamic acid 5-benzyl ester; Figure S2: NMR spectrum of N-benzyloxycarbonyl-L-thioglutamic acid 5-benzyl ester; Figure S3: NMR spectrum of L-thioglutamic acid; Table S1: Statistical analysis using Tukey’s HSD test for data presented in Figure 1 between different samples at the same time points. Table S2: Statistical analysis using Tukey’s HSD test for data presented in Figure 1 and between the same samples at different time points. Table S3. Statistical analysis using Tukey’s HSD test for data presented in Figure 2 and Figure 3 between different samples at the same time points. Table S4. Statistical analysis using Tukey’s HSD test for data presented in Figure 2 and Figure 3 between the same samples at different time points. Table S5. Statistical analysis using Tukey’s HSD test for data presented in Figure 6 and Figure 7 between different samples at the same time points. Table S6. Statistical analysis using Tukey’s HSD test for data presented in Figure 6 and Figure 7 between the same samples at different time points. Groups that possess different letters have statistically significant differences (*p* < 0.05) in mean whereas those that possess the same letter are statistically similar.
