# Supplementary material for: Hydrogen Sulfide Delivery to Enhance Bone Tissue Engineering Cell Survival
Source: Pharmaceuticals (Basel). 2024 May 2;17(5):585. doi: 10.3390/ph17050585 (PMC11124412; doi:10.3390/ph17050585)
Supplement: Supplementary file 1 [file pharmaceuticals-17-00585-s001.zip › pharmaceuticals-2896923-supplementary.pdf]

Supplementary Information

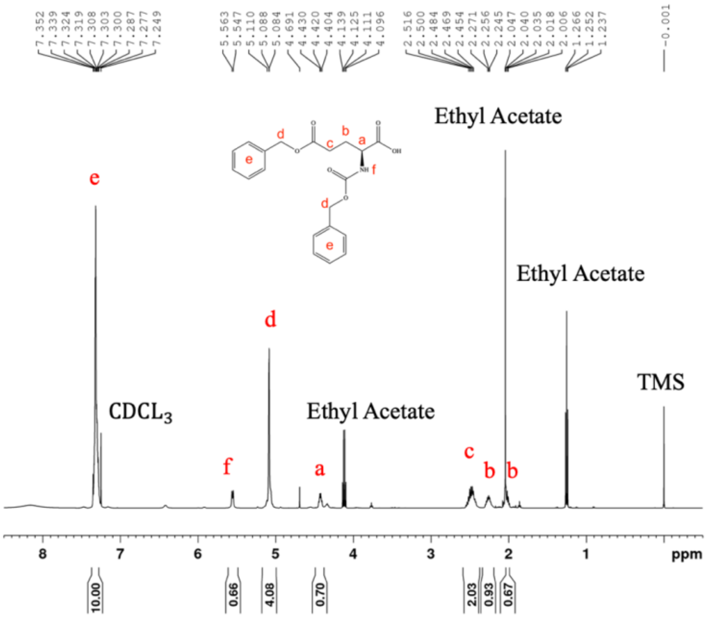

Figure S1. NMR spectrum of N-benzyloxycarbonyl-L-glutamic acid 5-benzyl ester.

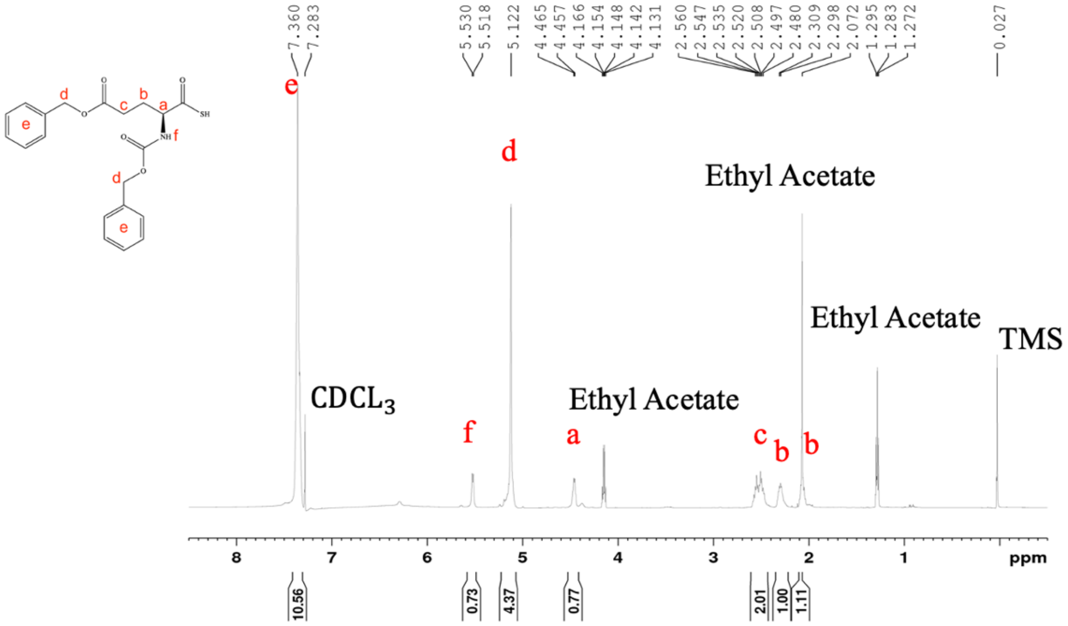

Figure S2. NMR spectrum of N-benzyloxycarbonyl-L-thioglutamic acid 5-benzyl ester.

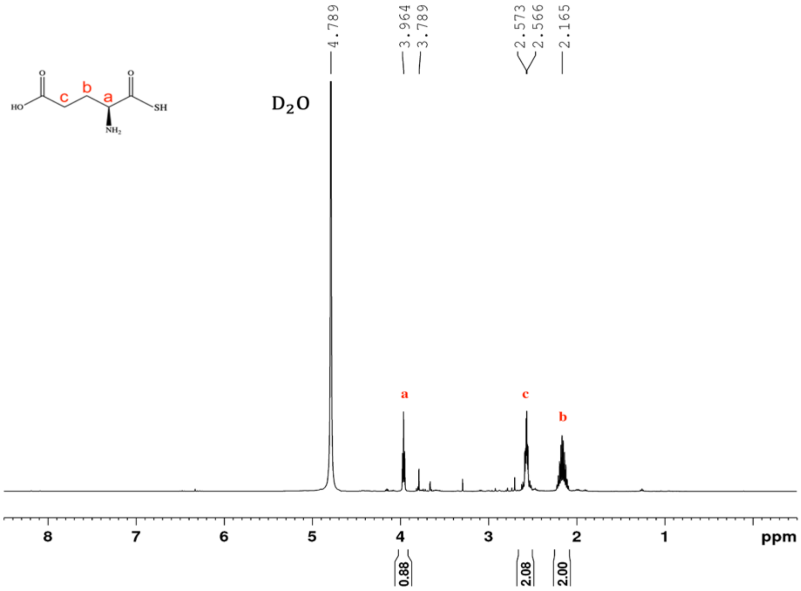

Figure S3. NMR spectrum of L-thioglutamic acid.

Table S1. Statistical analysis using Tukey's HSD test for data presented in **Figure 1** between different samples at the same time points. Groups that possess different letters have statistically significant differences ($p < 0.05$) in mean whereas those that possess the same letter are statistically similar.

NaSH	Cell Number	MTS
Day 1		
Ctrl	A	
0.25 mM	AB	BCD
0.5 mM	ABC	ABCD
1 mM	ABC	D
2 mM	ABC	CD
4 mM	ABC	ABCD
8 mM	CD	A
16 mM	CD	ABC
32 mM	BCD	AB
64 mM	CD	AB
Day 3		
Ctrl	AB	
0.25 mM	A	A
0.5 mM	A	A
1 mM	A	A
2 mM	A	A
4 mM	A	A
8 mM	BC	A
16 mM	C	A
32 mM	C	A
64 mM	C	A
Day 7		
Ctrl	AB	
0.25 mM	A	A
0.5 mM	A	A
1 mM	A	A

2 mM	AB	A
4 mM	AB	A
8 mM	BC	A
16 mM	C	A
32 mM	C	A
64 mM	C	A
Day 14		
Ctrl	B	
0.25 mM	AB	ABC
0.5 mM	A	ABCD
1 mM	A	ABCD
2 mM	AB	ABCD
4 mM	AB	AB
8 mM	C	A
16 mM	CD	ABCD
32 mM	C	CD
64 mM	DE	BCD

Table S2. Statistical analysis using Tukey's HSD test for data presented in **Figure 1** and between the same samples at different time points. Groups that possess different letters have statistically significant differences ($p < 0.05$) in mean whereas those that possess the same letter are statistically similar.

**NaSH**	**Cell Number**	**MTS**
Ctrl		
Day 1	Z	
Day 3	Y	
Day 7	X	
Day 14	W	
0.25 mM		
Day 1	Z	Z
Day 3	Y	Z
Day 7	Y	Z
Day 14	X	Z
0.5 mM		
Day 1	Z	Z
Day 3	Y	Z
Day 7	Y	Z
Day 14	X	Z
1 mM		
Day 1	Z	Z
Day 3	YZ	Z
Day 7	Y	Z
Day 14	X	Z
2 mM		
Day 1	Z	Z
Day 3	Y	Z
Day 7	Y	Z
Day 14	X	Z
4 mM		
Day 1	Z	Z
Day 3	Y	Z

Day 7	Y	Z
Day 14	X	Z
8 mM		
Day 1	Z	Z
Day 3	Y	Z
Day 7	Y	Z
Day 14	X	Z
16 mM		
Day 3	Z	Z
Day 7	Z	Z
Day 14	Z	Z
Day 21	Y	Z
32 mM		
Day 1	Z	Z
Day 3	Z	YZ
Day 7	Z	Y
Day 14	Y	Y
64 mM		
Day 3	Z	Y
Day 7	YZ	YZ
Day 14	XY	Y
Day 21	X	Y

Table S3. Statistical analysis using Tukey's HSD test for data presented in **Figure 2** and **Figure 3** between different samples at the same time points. Groups that possess different letters have statistically significant differences ($p < 0.05$) in mean whereas those that possess the same letter are statistically similar.

	Cell Number	MTS	ALP	ALZ
Day 1				
Ctrl	A		C	A
Ca_{32}	C	C	B	A
P_{16}	C	D	B	A
Ca_{32}/P_{16}	C	CD	AB	A
$NaSH_1$	AB	B	B	A
$Ca_{32}/NaSH_1$	BC	AB	AB	A
$P_{16}/NaSH_1$	BC	AB	A	A
$Ca_{32}/P_{16}/NaSH_1$	BC	A	A	A
Day 3				
Ctrl	B		D	AB
Ca_{32}	D	B	C	A
P_{16}	D	B	C	AB
Ca_{32}/P_{16}	D	B	C	B
$NaSH_1$	A	A	D	B
$Ca_{32}/NaSH_1$	C	A	AB	B
$P_{16}/NaSH_1$	C	A	B	AB
$Ca_{32}/P_{16}/NaSH_1$	C	A	A	B
Day 7				
Ctrl	A		D	C
Ca_{32}	BC	D	C	C
P_{16}	BC	D	C	C

Ca_{32}/P_{16}	C	D	C	C
$NaSH_1$	A	C	D	C
$Ca_{32}/NaSH_1$	B	B	AB	AB
$P_{16}/NaSH_1$	B	B	B	B
$Ca_{32}/P_{16}/NaSH_1$	BC	A	A	A
Day 14				
Ctrl	B		F	D
Ca_{32}	D	B	E	D
P_{16}	D	B	E	D
Ca_{32}/P_{16}	D	B	D	D
$NaSH_1$	A	A	F	D
$Ca_{32}/NaSH_1$	C	A	C	C
$P_{16}/NaSH_1$	C	A	B	B
$Ca_{32}/P_{16}/NaSH_1$	C	A	A	A

Table S4. Statistical analysis using Tukey's HSD test for data presented in **Figure 2** and **Figure 3** between the same samples at different time points. Groups that possess different letters have statistically significant differences ($p < 0.05$) in mean whereas those that possess the same letter are statistically similar.

	**Cell Number**	**MTS**	**ALP**	**ALZ**
Ctrl				
Day 1	Z		Z	Z
Day 3	Y		Z	Z
Day 7	X		Z	Z
Day 14	X		Z	Z
Ca_{32}				
Day 1	Z	Z	Z	Z
Day 3	Z	Y	Z	Z
Day 7	Z	X	Y	Z
Day 14	Z	W	X	Z
P_{16}				
Day 1	Z	Z	Z	Z
Day 3	Z	Y	Y	Z
Day 7	Z	Y	Y	Z
Day 14	Z	X	X	Z
Ca_{32}/P_{16}				
Day 1	Z	Z	Z	Z
Day 3	YZ	Y	Y	Z
Day 7	YZ	X	X	Z
Day 14	Y	W	W	Y
$NaSH_1$				
Day 1	Z	Z	Z	Z
Day 3	Y	Z	Y	Z
Day 7	Y	Z	XY	Z
Day 14	X	Z	X	Z
$Ca_{32}/NaSH_1$				
Day 1	Z	Z	Z	Z
Day 3	YZ	Z	Z	Z
Day 7	Y	Z	Y	Y
Day 14	X	Z	X	X

$P_{16}/NaSH_1$				
Day 1	Z	Z	Z	Z
Day 3	YZ	Z	Z	Z
Day 7	Y	Z	Y	Y
Day 14	X	Z	X	X
$Ca_{32}/P_{16}/NaSH_1$				
Day 1	Z	Z	Z	Z
Day 3	YZ	Z	Z	Z
Day 7	Y	Z	Y	Y
Day 14	X	Z	X	X

Table S5. Statistical analysis using Tukey's HSD test for data presented in **Figure 6** and **Figure 7** between different samples at the same time points. Groups that possess different letters have statistically significant differences ($p < 0.05$) in mean whereas those that possess the same letter are statistically similar.

	**Cell Number**	**MTS**	**ALP**	**ALZ**
Day 1				
Ctrl	A		FG	AB
Ca_{32}	BCDE	C	EF	AB
P_{16}	BCDE	E	DE	AB
Ca_{32}/P_{16}	CDE	CDE	CDE	AB
Glu_{32}	E	CDE	BCDE	AB
Ca_{32}/Glu_{32}	CDE	CDE	DE	AB
P_{16}/Glu_{32}	DE	CD	BCD	AB
$Ca_{32}/P_{16}/Glu_{32}$	E	DE	ABC	A
$GluSH_{32}$	BCD	B	G	B
$Ca_{32}/GluSH_{32}$	BC	AB	CDE	A
$P_{16}/GluSH_{32}$	AB	A	AB	AB
$Ca_{32}/P_{16}/GluSH_{32}$	BCDE	AB	A	A
Day 3				
Ctrl	A		E	AB
Ca_{32}	C	BC	D	AB
P_{16}	C	D	C	AB
Ca_{32}/P_{16}	C	BCD	CD	B
Glu_{32}	C	BCD	C	AB
Ca_{32}/Glu_{32}	C	BCD	C	AB
P_{16}/Glu_{32}	C	BCD	BC	AB
$Ca_{32}/P_{16}/Glu_{32}$	C	CD	AB	A
$GluSH_{32}$	A	B	E	AB
$Ca_{32}/GluSH_{32}$	B	A	A	AB
$P_{16}/GluSH_{32}$	B	A	A	AB
$Ca_{32}/P_{16}/GluSH_{32}$	B	A	A	AB
Day 7				
Ctrl	A		F	C
Ca_{32}	C	DE	DE	C
P_{16}	C	D	E	C
Ca_{32}/P_{16}	C	D	DE	C
Glu_{32}	C	E	CD	C
Ca_{32}/Glu_{32}	C	DE	CDE	C
P_{16}/Glu_{32}	C	D	CDE	C

$Ca_{32}/P_{16}/Glu_{32}$	C	DE	C	C
$GluSH_{32}$	A	C	F	C
$Ca_{32}/GluSH_{32}$	B	B	B	B
$P_{16}/GluSH_{32}$	B	A	B	AB
$Ca_{32}/P_{16}/GluSH_{32}$	B	B	A	A
Day 14				
Ctrl	A		F	D
Ca_{32}	C	C	CDE	D
P_{16}	C	C	CD	D
Ca_{32}/P_{16}	C	C	C	D
Glu_{32}	C	C	E	D
Ca_{32}/Glu_{32}	C	C	DE	D
P_{16}/Glu_{32}	C	C	CD	D
$Ca_{32}/P_{16}/Glu_{32}$	C	C	C	D
$GluSH_{32}$	A	B	F	D
$Ca_{32}/GluSH_{32}$	B	B	B	C
$P_{16}/GluSH_{32}$	B	A	B	B
$Ca_{32}/P_{16}/ GluSH_{32}$	B	AB	A	A

Table S6. Statistical analysis using Tukey's HSD test for data presented in **Figure 6** and **Figure 7** between the same samples at different time points. Groups that possess different letters have statistically significant differences ($p < 0.05$) in mean whereas those that possess the same letter are statistically similar.

	**Cell Number**	**MTS**	**ALP**	**ALZ**
Ctrl				
Day 1	Z		Z	Z
Day 3	Y		Z	Z
Day 7	X		Z	Z
Day 14	X		Z	Z
Ca_{32}				
Day 1	Z	Z	Z	Z
Day 3	Z	Y	Z	Z
Day 7	Z	X	Y	Z
Day 14	Z	W	X	Z
P_{16}				
Day 1	Z	Z	Z	Z
Day 3	Z	Y	Y	Z
Day 7	Z	Y	Y	Z
Day 14	Z	X	X	Z
Ca_{32}/P_{16}				
Day 1	Z	Z	Z	Z
Day 3	YZ	Y	Y	Z
Day 7	YZ	X	X	Z
Day 14	Y	W	W	Y
Glu_{32}				
Day 1	Z	Z	Z	Z
Day 3	YZ	Y	Z	Z
Day 7	YZ	X	Z	Z
Day 14	Y	W	Z	Z
Ca_{32}/Glu_{32}				

Day 1	Z	Z	Z	Z
Day 3	Z	Y	Y	YZ
Day 7	Z	X	X	YZ
Day 14	Z	W	W	Y
P_{16}/Glu_{32}				
Day 1	Z	Z	Z	Z
Day 3	Z	Y	Y	YZ
Day 7	Z	X	Y	YZ
Day 14	Z	W	X	Y
Ca_{32}/P_{16}/Glu_{32}				
Day 1	Z	Z	Z	Z
Day 3	YZ	Y	Y	YZ
Day 7	YZ	X	X	YZ
Day 14	Y	W	W	Y
$GluSH_{32}$				
Day 1	Z	Z	Z	Z
Day 3	Y	Z	YZ	YZ
Day 7	Y	Z	Y	YZ
Day 14	X	Z	Y	Y
Ca_{32}/$GluSH_{32}$				
Day 1	Z	Z	Z	Z
Day 3	YZ	Z	Y	Z
Day 7	Y	Z	X	Y
Day 14	X	Z	W	W
P_{16}/$GluSH_{32}$				
Day 1	Z	Z	Z	Z
Day 3	YZ	Z	Y	Z
Day 7	Y	Z	X	Y
Day 14	X	Z	W	W
Ca_{32}/P_{16}/$GluSH_{32}$				
Day 1	Z	Z	Z	Z
Day 3	YZ	Z	Y	Z
Day 7	Y	Z	X	Y
Day 14	X	Z	W	W
